# Survivin, a novel target of the Hedgehog/GLI signaling pathway in human tumor cells

**DOI:** 10.1038/cddis.2015.389

**Published:** 2016-01-14

**Authors:** K Vlčková, L Ondrušová, J Vachtenheim, J Réda, P Dundr, M Zadinová, P Žáková, P Poučková

**Affiliations:** 1 Laboratory of Transcription and Cell Signaling, Institute of Medical Biochemistry and Laboratory Diagnostics, Charles University in Prague, 1st Faculty of Medicine, Prague, Czech Republic; 2 Institute of Pathology, Charles University in Prague, 1st Faculty of Medicine and General University Hospital in Prague, Prague, Czech Republic; 3 Institute of Biophysics and Informatics, Charles University in Prague, 1st Faculty of Medicine, Prague, Czech Republic

## Abstract

Survivin, an important antiapoptotic protein, is expressed in tumors, whereas in normal tissues the expression of this protein is extremely low, defining a role for survivin as a cancer gene. Survivin exhibits multifunctional activity in tumor cells. However, why survivin expression is sharply and invariably restricted to tumor tissue remains unclear. Here, we identified 11 putative consensus binding sites for GLI transcription factors in the survivin promoter and characterized the promoter activity. Inhibitors of the Hedgehog/GLI pathway, cyclopamine and GANT61, decreased the promoter activity in reporter assays. ΔNGLI2 (which lacks the repressor domain) was the most potent vector in activating the survivin promoter–reporter. Moreover, GANT61, a GLI1/2 inhibitor, repressed endogenous survivin protein and mRNA expression in most cells across a large panel of tumor cell lines. Chromatin immunoprecipitation showed GLI2 binding to the survivin promoter. The ectopic GLI2-evoked expression of endogenous survivin was observed in normal human fibroblasts. GANT61 decreased survivin level in nude mice tumors, mimicking the activity of GANT61 in cultured cells. The immunohistochemistry and double immunofluorescence of human tumors revealed a correlation between the tissue regions showing high GLI2 and survivin positivity. Thus, these results demonstrated that survivin is a classical transcriptional target of GLI2, a Hedgehog pathway signaling effector. This potentially reflects the high expression of survivin in human tumor cells. As the Hedgehog pathway is upregulated in virtually all types of cancer cells, these findings substantially contribute to the explanation of uniform survivin expression in tumors as a potential target for the development of a more effective treatment of cancers through the inhibition of GLI2 to restrain survivin activity.

Survivin is a single-baculovirus IAP repeat protein that plays a role in multiple processes, including proliferation and cell survival. Survivin is abundantly and ubiquitously expressed during development^1^ and this expression is consistently recapitulated in tumor tissue. The expression of this protein has been associated with the aggressive biological features of tumors, resistance to radiation and chemotherapy and poor clinical outcome.^2^ Since its discovery in 1997,^3^ the mechanism that maintains high survivin expression in tumors and absent or extremely low survivin expression in normal tissues remains unknown. It has been suggested that the basal transcription of the survivin gene is primarily regulated through the Sp family of transcription factors.^4, 5^ Although binding sites for several pro-oncogenic transcription factors (Sp1, STAT3, NF-kB, KLF5, E2F1, DEC1 or TCF) are present in the survivin promoter (reviewed by Boidot *et al.*^6^) and might be important in the elevation of survivin levels, specifically in individual tumors, it is unlikely that these sites could guarantee the high survivin levels observed in all malignant cells. For example, KLF5 increased the resistance of ovarian cancer cells to drug treatment,^7^ and DEC1 increased the expression of survivin via Sp1 sites in kidney and lung adenocarcinomas.^8^ A recent study reported that HH/GLI via GLI1 and GLI2 transcription factors activated the E2F1 promoter in melanoma cells, and E2F1 contributed to the increase of melanoma cell growth,^9^ constituting a positive feedback loop.

The canonical activation of the Sonic Hedgehog (HH/GLI) cascade involves the binding of the ligand (Shh) to the 12-pass membrane protein PATCHED (PTCH), which releases the activity of adjacent 7-pass transmembrane protein Smoothened (SMO). Subsequently, the effector proteins GLI(1–3) are released from the inhibitor SuFu (Suppressor of Fused) and translocated to the nucleus to activate target genes.^10^ HH/GLI is important during normal embryonic development, and the aberrant activation of this signaling pathway has been associated with many human cancers. The activation of HH/GLI increases proliferation and survival, induces cancer stem cell marker expression and enhances bone metastasis.^11, 12^

Several studies have implicated a non-canonical Hh signaling pathway in regulating HH/GLI signaling, thus substituting the necessity of upstream ligand signaling.^13, 14, 15, 16^ Several pathways, such as AKT,^17, 18, 19^ MAPK,^18^ RAS^20^ or EGFR,^21, 22^ can activate GLI factors in tumor cells. Conversely, many critical cellular pathways, such as proliferation, DNA damage repair, apoptosis, autophagy, epithelial–mesenchymal transition (EMT), telomerase activity, invasion, metastasis and maintenance of tumor stem cells, are regulated through GLI transcription factors.^23, 24, 25, 26, 27^ In recent years, a specific and effective GLI1/2 inhibitor GANT61 has been successfully used for the *in vitro* and *in vivo* treatment of cancer cells containing either the canonical or non-canonical activation of HH/GLI.^28, 29^

Here, we show that survivin is a transcriptional target of the Hedgehog pathway effector factor GLI2, and harbors 11 potential GLI-binding sites in the promoter. GLI2 is a pro-invasive protein present in most tumor cell lines and this protein could substantially contribute to the stably elevated survivin levels observed in tumors. We further demonstrated the binding of GLI2 to the survivin promoter and the decreased expression of survivin protein and RNA after treatment with GLI2 inhibitor GANT61 in a large panel of tumor cell types. Furthermore, endogenous survivin expression is evoked through the ectopic expression of GLI2 in normal human fibroblasts. Overall, the results of the present study suggest that survivin is a novel target of the Hedgehog/GLI pathway and GLI2 is the primary upregulating factor for this protein. Thus, the maintenance of deregulated survivin expression in many tumors could reflect activated Hedgehog pathway.

## Results

### Activities of the survivin promoter–reporter with 11 potential GLI-binding sites determined in A549 cells

**Figure 1:**
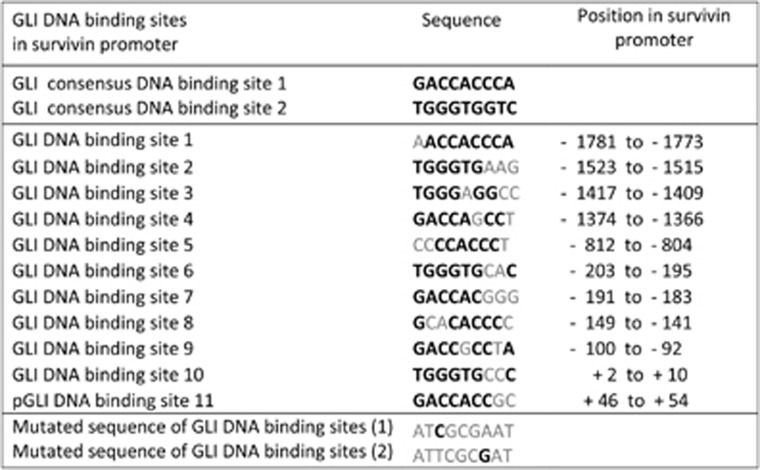
Overview of the GLI-binding sites and their position in the human survivin promoter. Numbering is from initiating ATG (+1). Full consensus sites and the sequence used for introduced mutations is also given. Black letters are consensus nucleotides, light letters are non-consensus nucleotides. Survivin-containing GLI sites are degenerated in 1–3 nucleotides. GLI site positions are shown in Figure 2

We reasoned that the high expression of the cancer protein survivin, invariably present in all tumor cells, should have a more significant impact than anticipated. The presence of several pro-oncogenic sites in the promoter presumably cannot explain the universal expression of this protein in tumors (for promoter map, see Boidot *et al.*^6^). We observed that the survivin promoter contains 11 sites for binding GLIs, effectors of the HH/GLI signaling pathway; however, none of these sites are full consensus sequences (Figure 1). Non-consensual sites with two or three mismatches can still activate the transcription of other GLI-regulated genes, such as BCL2 and osteopontin.^30, 31^ Thus, the survivin promoter contains attributes for the binding and eventual activation by GLI transcription factors.

**Figure 2:**
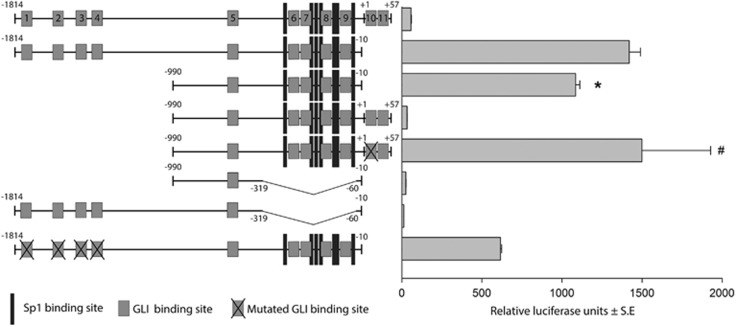
Schematic representation of the survivin promoter with the numbering (relative to translation start) and indicated GLI-binding sites (no. 1–11). The GLI sites correspond to those depicted in Figure 1. The positions of Sp-binding sites are also designated. To determine the luciferase values, the promoters cloned in pGL3basic (Promega) were transfected into A549 cells by using LipoJet (SignaGen) (1 *μ*g per well in a 12-well plate). Dual luciferase system (Promega) was utilized for detection. Each sample was transfected in triplicates and three repeated experiments were performed, in which similar results were obtained. S.E. values are shown for all luciferase results. *, statistically significant (*P*<0.05); #, statistically not significant (related to the −1814−10 promoter)

To determine the activity of the survivin promoter and elucidate the importance of the GLI sites, we transfected several versions of the promoter into A549 cells and measured luciferase activity (Figure 2). The proximal promoter (−990 to −10) was sufficient to activate luciferase activity and was only slightly less efficient than the longer (−1814−10) promoter. The distal portion of the promoter contains four GLI sites (no. 1–4), and the mutations at these sites appreciably decrease promoter activity (~2.5-fold; Figure 2). We individually mutated all 11 sites, but no single mutation (with the exception of site no. 10) had a significant effect on the promoter activity (data not shown). Intriguingly, after the addition of a short downstream region (−10 to +57 nt) containing sites 10 and 11, the strong repression of promoter activity appeared. However, when site 10 was mutated the activity was reverted to normal, indicating that the site 10 is an inhibitory site. Of note, as survivin is widely expressed in tumors, this site cannot be necessarily repressory in the genomic context, where transcription occurs differently than in reporter assays.

To further precisely define the regulatory regions of the survivin promoter, we deleted the central region of the proximal promoter (−390 to −60) (Figure 2). After the deletion of this region in both the longer and shorter survivin promoters, the activity was completely abrogated. Because this region comprises all Sp sites, this result is consistent with the known role of Sp1 and Sp3 as factors necessary for the basic promoter activity.^4, 5^ The role of GLI sites no. 6–9 in the deleted region cannot be precisely established because some of these areas overlap the Sp sites. Taken together, these results suggest that the promoter and regulation of survivin through GLI sites is complex, with one site (no. 10) being clearly inhibitory in reporter assays, and the proximal region, containing four GLI sites, and the central region with Sp sites (−390 to −60) are required for the transcriptional activity of the promoter. Most likely, a specific combination of some of the sites is responsible for the full reporter activity. Moreover, the four distal sites increase survivin promoter activity.

### The survivin promoter–reporter is inhibited through Hedgehog/GLI inhibitors cyclopamine and GANT61 and specifically activated through ΔNGLI2

**Figure 3:**
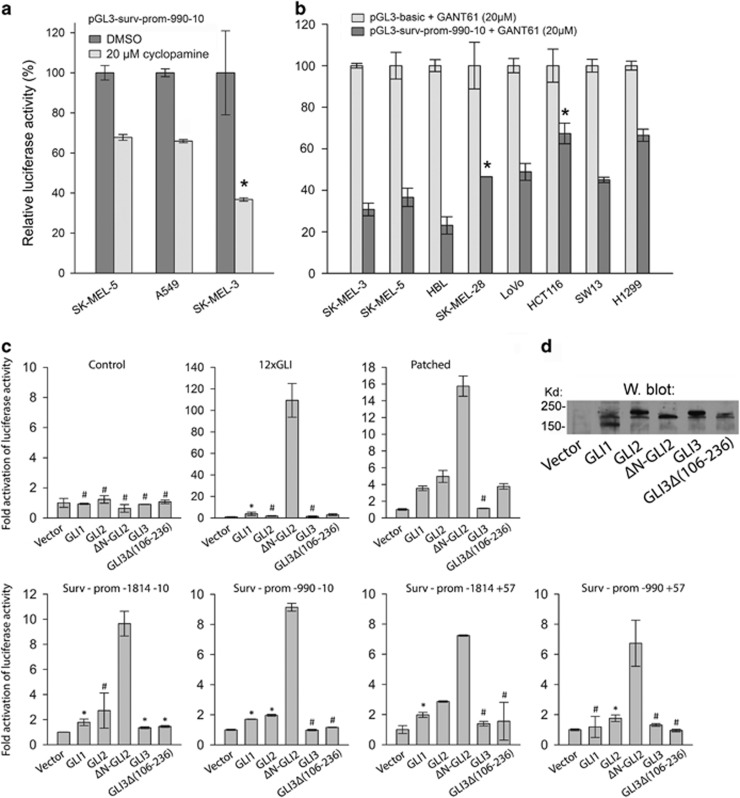
Inhibition of survivin promoter by cyclopamine and GANT61 and survivin promoter stimulation by GLI factors in A549 cells. (**a**) Cyclopamine inhibits luciferase activity in the three melanoma cell lines after 24 h treatment. (**b**) Survivin promoter activities are inhibited after 24 h incubation with GANT61 in eight tumor cell lines. GANT61 has a minimal effect on control promoter (usually <5%). Therefore, control values are set as 100% (control plasmid with GANT61). (**c**) Stimulation of several promoters with GLI1, GLI2, ΔNGLI2, GLI3 and GLI3Δ (106–236). Upper row: the activities of the control promoters are similar, an artificial 12xGLI promoter and a known GLI target PATCHED promoter are strongly activated by ΔNGLI2. Lower row: the four indicated survivin promoter versions are activated, albeit sometimes weakly, by all three GLIs, maximally by ΔNGLI2 (from 7- to 10-fold). GLI3 displayed no activation of survivin promoters, while higher activity was noted with GLI3Δ(106–236). Please note that the control values of survivin promoters are set as 1 and the amount of their DNA is fivefold lower than in Figure 2, but the controls still have relatively high activity, as documented in RLU in Figure 2. Each experiment was repeated at least two times (**d**) Western blot demonstrating the expression of GLI factors expressed in (**c**). All GLI proteins were N-terminal FLAG-tagged constructs and detected through the anti-FLAG antibody

Given that many GLI sites are present in the survivin promoter, inhibitors of the HH/GLI pathway should suppress promoter activity. Indeed, a SMO inhibitor cyclopamine significantly diminished the activity of the survivin promoter in many cell lines to various extents (30–70%), as exemplified in three melanoma cell lines (Figure 3a). We next assessed the inhibition of HH/GLI using a downstream inhibitor GANT61, which specifically inhibits GLI1/2 activity. In eight tested cell lines, GANT61 consistently inhibited the promoter, with only 70–20% of the original activity remaining (Figure 3b). The effect of cyclopamine and GANT61 was dose dependent (Supplementary Figure S1). These results suggest that survivin expression is regulated through the HH/GLI pathway.

To further confirm the transcriptional regulation of survivin through HH/GLI signaling, we compared the activity of four types of survivin promoters with the known GLI targets, the 12xGLI promoter^32^ and the PATCHED promoter.^33^ An empty vector (control) or expression FLAG-tagged vectors for GLI1, GLI2, ΔNGLI2 (an active form of GLI2)^34^, GLI3 and GLI3Δ(106–236) were cotransfected into A549 cells. Among all promoters, ΔNGLI2 exhibited the highest activation potential. The super-GLI promoter 12xGLI was activated nearly 100-fold and the PATCHED promoter 16-fold through ΔNGLI2 (Figure 3c, upper row). The survivin promoters were stimulated seven- to ninefold (Figure 3c, lower row). GLI1 and GLI3 did not activate the survivin promoters. GLI3 is a known repressor of some HH/GLI-regulated genes).^35, 36^ Stimulation through GLI3Δ(106–236), which lacks the repressor domain, was consistently higher than that of GLI3 (Figure 3c, lower row). The most prominent activation of all promoters was through ΔNGLI2. The activation by GLI2, ΔNGLI2 and GLI3Δ(106–236) were dose dependent (Supplementary Figure S2). All GLI proteins were expressed, as assessed by the anti-FLAG antibody (Figure 3d).

### GANT61 inhibits survivin protein and mRNA expression in tumor cells

**Figure 4:**
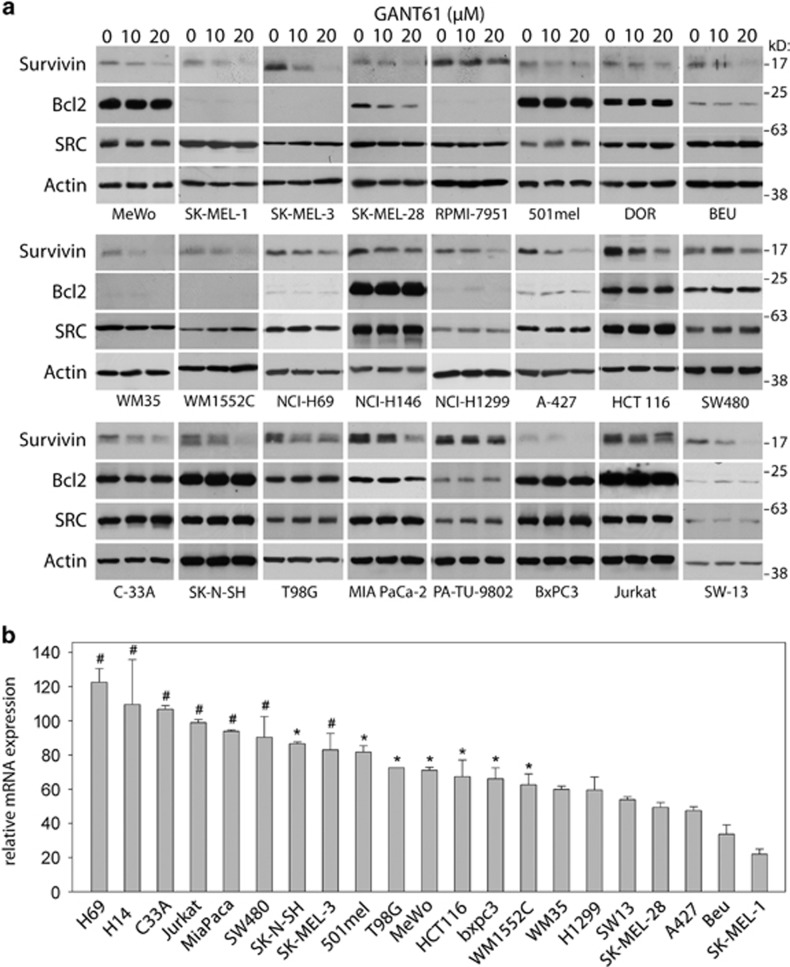
Inhibition of the survivin cellular protein and its mRNA expression after GANT61 treatment. (**a**) Cells were treated with indicated GANT61 concentrations for 24 h. RIPA buffer extracts were prepared, blotted and probed with the indicated antibodies. Additional cell lines with no or only minute changes of survivin protein expression are shown in Supplementary Figure S3 (**b**) Real-time PCR experiments performed from controls and samples inhibited by 20 *μ*M GANT61. After 24 h in GANT61, the RNA was isolated with Trizol, reverse transcribed and used in qPCR. Distinct decrease of RNAs levels was observed relative to controls (0 *μ*M GANT61). RNA diminutions do not correlate neither with the tumor cell type nor with the proliferation rate. Some tumor cells produced only slightly reduced RNA, whereas the survivin protein diminished strikingly (e.g., SK-N-SH and SK-MEL-3), suggesting that the protein degradation was retarded as well (see text and Supplementary Figure S6). Knockdown of GLI2 RNA by shRNA plasmid constructs in MeWo and SW13 cell lines also diminished survivin mRNA and protein expression (not shown). #, not significant; *, significant at *P*<0.05; not marked, significant at *P*<0.01 (relative to 100%)

Instead of studying the HH/GLI target survivin in a specific tumor, we used a general approach to determine a potentially more common role for GLIs in endogenous survivin expression in several tumor cell types. The consequence of GLI factors inhibition was estimated across a large panel of cancer cell lines (total 40 lines, listed in Supplementary Table S1). Although approximately one-half of the cell lines were melanomas or small-cell lung carcinomas, several other malignant cell types, such as NSCLC, colon and pancreatic cancers, were also represented. Western blot analysis revealed that GANT61 inhibited or attenuated survivin expression in most cell lines (Figure 4a) in a dose-dependent manner. GANT61 remained on the cells for 24 h at 0, 10 or 20 *μ*M and the RIPA extracts were analyzed. Marked differences in the extent of decreased survivin expression were observed. In some cell lines survivin expression was dramatically decreased (SK-MEL-3, WM-35, SW13), particularly with 20 *μ*M GANT61, and some cells responded weakly. There was no change in expression of the controls SRC and actin. The differences in the decreased survivin protein expression did not correlate with the type of tumor. In some cell lines, no or minimal changes in survivin protein expression were detected after GANT61 treatment (Supplementary Figure S3). Regl *et al.*^30^ demonstrated that BCL2 transcription is activated through GLI2. In the present study, we observed that BCL2 was downregulated through GANT61 in only one cell line, SK-MEL-28 (Figure 4a). Osteopontin (OPN) has previously been identified as a target of HH/GLI signaling in melanoma cells.^31^ In the present study, however, we did not detect a decrease in OPN expression after GANT61 treatment in any cell line (data not shown).

Consistently, real-time PCR revealed that the inhibition of survivin protein levels was accompanied with the downregulation of survivin mRNA to various extents (Figure 4b), confirming the predominantly transcriptional repression of this gene after GANT61 treatment (both parameters correlated, albeit weakly; Supplementary Figure S4). We also estimated correlation of quantified values of GLI2 and survivin proteins from western blots, but these two parameters did not correlate (Supplementary Figure S5). This is not surprising because GLI2 has many targets in the cell and both low and high survivin protein levels were responsive to GANT61. In some cell lines (e.g., SK-MEL-3), the decline in RNA levels was apparently lower than the decrease in protein levels (Figure 4a). We hypothesized that the rate of survivin protein degradation might also be accelerated or delayed in some cell lines. Thus, four cell lines were randomly selected and incubated with GANT61 for 24 h and MG132 (MG), a proteasome inhibitor, was added to one sample for the last 6 h to prevent survivin degradation (Supplementary Figure S6). In SK-MEL-3 cells (high RNA), MG did not overcome the diminution of the GANT61-mediated survivin protein level, indicating that some GLI factors also protect survivin protein from degradation. In A427 cells, the decreased survivin levels reflect decreased RNA expression. The 501mel cells (high RNA) reacted poorly to GANT61 treatment and were not influenced after incubation with MG. However, in H1299 cells, MG increased the final levels of survivin protein, indicating that the protein protection is mediated through an as yet unknown, GLI-independent mechanism. Taken together, these results suggest that the decrease in endogenous survivin in the presence of GANT61 is predominantly a transcriptional event, whereas the specific cellular context might also differently modify protein degradation.

### GLI2 binds to the survivin promoter in cells

**Figure 5:**
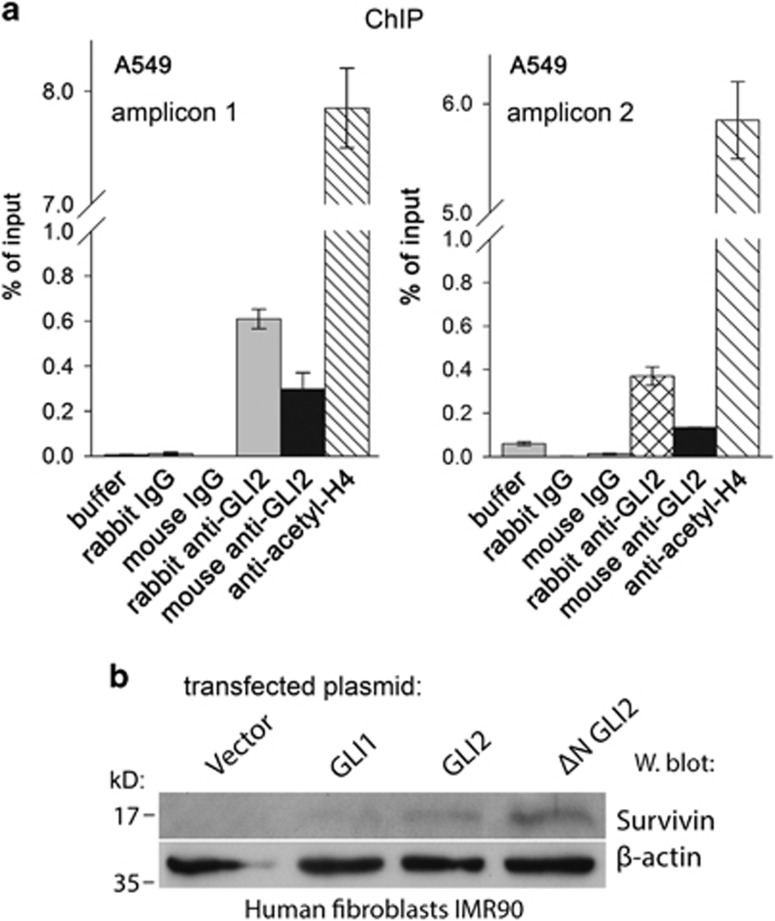
Chromatin immunoprecipitation and endogenous survivin induction by GLI2 in IMR90 fibroblasts. (**a**) A549 cells transfected with ΔNGLI2 expression vector were chromatin immunoprecipitated and resulting DNA was subjected to quantification by qPCR. All negative controls (buffer, mouse or rabbit non-immune IgG) and a positive control (antibody against acetylated histone H4) were included. Significant enrichment of GLI2 samples was evident in both amplifications. The two real-time PCRs differed only in the antisense primer. The amplicon shown in (**a**) on the right was shorter. (**b**) Transient transfection of GLI1, GLI2 and ΔNGLI2 into human IMR90 fibroblasts, which normally do not express survivin and GLI2, induced expression of survivin protein (about 30% cells were transfected, not shown). Most prominent expression of survivin protein was achieved with ΔNGLI2, which was also most active in reporter assays (Figure 3). Western blot with the anti-GLI2 antibody verified the GLI2 increase in transfected cells. The results indicate the dependence of survivin expression on GLI2 in normal cells

Next, to confirm the direct interaction of GLI2 with the endogenous survivin promoter, we performed a quantitative chromatin immunoprecipitation assay (ChIP) in A549 cells using two different anti-GLI2 antibodies (Figure 5a). Preliminary experiments in several cell lines revealed only low (about twofold) enrichment of GLI2 on the promoter. Therefore, the ΔNGLI2 expressing plasmid was transfected into A549 cells, and after performing ChIP experiments, the GLI2 enrichment on the promoter was quantified using SYBR green real-time PCR. The results confirmed the prominent enrichment supporting that GLI2 was indeed recruited to the survivin promoter in cells. Similar qPCR results were obtained with two different amplifications resulting in two distinct amplicons (Figure 5a, left and right).

### Ectopic GLI2 elicits endogenous survivin expression in normal human fibroblasts

To further demonstrate that the survivin promoter is an actual GLI2 target in living cells, expression vectors for GLI1, GLI2 and ΔNGLI2 were transfected into the normal diploid human fibroblast cell line IMR90 which does not express survivin or GLI2. Western blotting clearly detected survivin in the resulting cell lysates. The strongest signal was obtained with the most potent activator ΔNGLI2 (Figure 5b), suggesting that GLI2 is a direct survivin promoter activator in cells. Ectopic GLI2 has been properly expressed (not shown).

### GANT61 partially inhibits tumor growth in GANT61-sensitive melanoma cell xenografts

*In vitro*, GANT61 completely killed SK-MEL-3 cells in 4 days, whereas 501mel cells were much more resistant (Supplementary Figure S7e). To determine whether the *in vitro* effects of GANT61 are recapitulated *in vivo*, SK-MEL-3 and 501mel cells were subcutaneously engrafted into athymic nude mice. While the tumor masses with GANT61 remained as controls in 501mel cells (Supplementary Figure S7b), two SK-MEL-3 tumors were markedly reduced (the size of one tumor deviated and did not diminish; Supplementary Figure S7a). Expectedly, in all tumors from GANT61-treated animals, survivin expression estimated by Western blot was markedly reduced (with the exception of the smallest 501mel tumor) (Supplementary Figures S7c and d). The immunochemistry of animal tumors showed positive survivin staining in controls, while only scarce positive cells remained in tumors from treated animals (Supplementary Figure S7f). Growth curves are also shown for all tumors (Supplementary Figures S7g and h).

### The immunochemically positive GLI2 and survivin regions in human tumor sections overlap and colocalize by immunofluorescence

**Figure 6:**
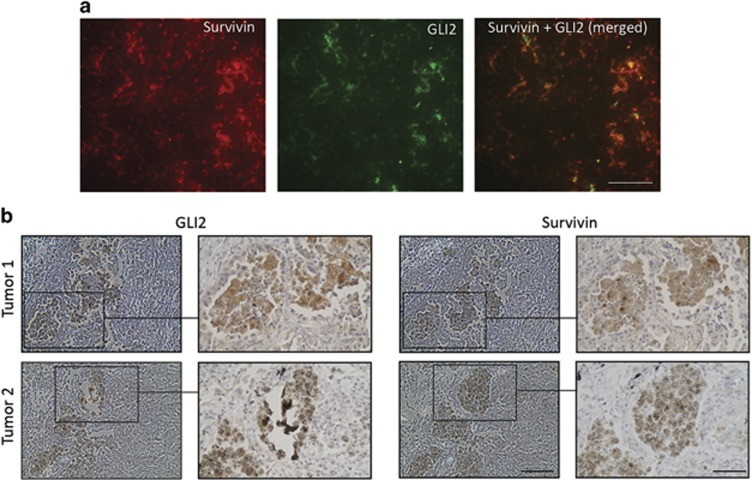
Immunohistochemical and immunofluorescence staining of GLI2 and survivin in human tumors. (**a**) Double immunofluorescence with anti-GLI2 (rabbit) and anti-survivin (mouse) antibodies demonstrating colocalization of both proteins in the areas of high staining activity of both. The staining has been performed on the frozen tissue (ovarian tumor) after acetone fixation. High correlation has been found using the FIJI software (Spearman's rank correlation value: 0.882). Bar represents 20 *μ*m. (**b**) Examples of two areas of lung tumors showing the highest score (4) of staining for survivin and GLI2. Parallel sections were stained. The same stained structures are clearly recognized in both lower and higher magnifications. Bars, 100 and 50 *μ*m (sectors)

Additional tests were performed to validate the role of GLI2 in survivin expression. If GLI2 is an activator of survivin transcription in cell models, then the positive areas for both proteins should correlate in authentic human tumor sections. We examined parallel sections of 35 randomly selected human tumors (lung carcinomas, ovary and tubal carcinomas and melanomas) and examined the GLI2 and survivin staining. Indeed, positively stained GLI2 areas correlated with survivin-positive areas in all tumors, although some regions of GLI2 positivity with survivin negativity and vice versa were also observed (not shown). The statistically significant positivity correlation was observed only in the highest score (4). Some tumor cells showed perfectly correlated positivity for both proteins in parallel sections, as exemplified in two cases of lung adenocarcinomas (Figure 6b). Apparently, strongly positive GLI2 cells were observed in parallel with sections showing positive survivin staining. To substantiate these results, double immunofluorescence was performed demonstrating colocalization of survivin and GLI in strongly staining areas in tumors. The simultanenous positivity of GLI2 and survivin images were highly statistically significant (Figure 6a), supporting the IHC results.

Thus, these data showed the overall correlation between GLI2 and survivin staining in tumors, strongly supporting that the HH/GLI pathway is an activator of survivin expression.

## Discussion

The Hedgehog pathway is important for multiple tumor types, although this signaling pathway was initially suggested as necessary for only basal cell carcinoma and medulloblastoma.^37^ Additionally, aberrant HH/GLI signaling plays a critical role in commonly occurring tumors, such as non-small-cell lung cancers^38^ and many others.^39^ However, the common mechanism for the maintenance of the high survivin levels in tumors remained obscure.

Several genes important for cancer growth are regulated through GLI factors. Previous studies have demonstrated the regulation of BCL2 via the HH pathway through GLI1,^40, 41^ while other studies have reported BCL2 activation through GLI2.^30^ Bar *et al.*^42^ reported that HH/GLI-activated BCL2 was important for the survival of medulloblastoma (a cancer not examined in the present study). However, in the present study, we observed BCL2 inhibition through GANT61 in only one cell line (Figure 4a). Conceivably, BCL2 might represent a HH/GLI target pivotal for tumor growth only in specific cell lines. Notably, even Sp factors, important for basal survivin promoter function, have also demonstrated pro-tumorigenic activity.^43^

The HH/GLI pathway functions in a paracrine and autocrine manner. Some inhibitors of HH/GLI have contributed to the downregulation of the pathway targets in the stromal microenvironment, suggesting that the effect on HH/GLI signaling is dependent on the stroma and a paracrine signaling mechanism.^44^ Owing to the critical role of this signaling pathway in general tumor maintenance, intensive clinical studies utilizing several HH/GLI inhibitors have been performed.^39, 45^

Here, we revealed a general mechanism resulting in survivin expression in cell lines. More than half of the cell lines analyzed showed downregulated survivin expression after treatment with 20 *μ*M GANT61, and several lines manifested nearly complete inhibition (Figure 4a). Further, higher doses of GANT61 efficiently inhibited survivin protein expression (data not shown), indicating the dose-dependent decrease and the reliance of survivin level on HH/GLI. Whereas some cells were resistant to the GANT61-mediated reduction of survivin expression (Supplementary Figure S3), other mechanisms likely maintain the survivin level observed in tumor cells. The survivin protein levels of GANT61-treated cells correlated with the real-time PCR values in samples where real-time PCR was performed (Supplementary Figure S4).

We dissected and mutated the survivin promoter, revealing many GLI-binding sites, and demonstrated that the promoter–reporter is substantially inhibited through the HH/GLI inhibitors cyclopamine and GANT61 (Figures 1–3). The large number of GLI-binding sites made it difficult to precisely determine the combination of sites critical for survivin activity mediated via HH/GLI. We propose that GLIs are associated with the promoter in each tumor cell and other factors dictate survivin expression. We previously demonstrated epigenetic mechanism important for survivin expression. The knockdown of BRG1, an ATPase of the SWI/SNF chromatin remodeling complex, dramatically reduced the expression of survivin RNA and protein in melanoma cells.^46^

Notably, both survivin and HH/GLI signaling are active during development. Survivin is lethal in homozygous knockout mice,^47^ similar to several components of the HH/GLI pathway (reviewed by Yang *et al.*^39^). Consistent with the present study, Brun *et al.*^48^ revealed that survivin is a critical therapeutic target in medulloblastoma cells, where HH/GLI signaling in invariably increased. The inhibition of survivin expression through the specific inhibitor YM155 profoundly affected the viability of tumor cells and sensitized tumors to radiation.

Taken together, we revealed the mysterious manner of expression of the tumor protein survivin, although the described mechanism is not functional in all tumor cell lines. These results show the direct activation of survivin expression through the HH/GLI signaling mediated by GLI2. Importantly, anti-GLI2 therapy or combined anti-GLI2 and anti-survivin therapies might decrease survivin in tumors and markedly improve the treatment of cancer.

## Materials and Methods

### Cell lines and treatments

We utilized 40 tumor cell lines of various origin (listed in Supplementary Table S1). Cell lines were cultivated in the cultivation media as recommended with 10% FCS and antibiotics. Most cell lines were from American Type Culture Collection (Manassas, VA, USA). Cell lines DOR, BEU, HBL, 501mel and PA-TU-8902 were from other sources (Supplementary Table S1). The cell lines were treated with inhibitors as indicated in the figures and figure legends (the final concentrations were 20 *μ*M GANT61 and 20 *μ*M cyclopamine).

### Quantitative real-time PCR

Total RNA was isolated using TRIZOL (Life Technologies, Carlsbad, CA, USA) according to the manufacturer's instructions. Total RNA (2 mg) was reverse transcribed using the Super Script II reverse transcriptase (Life Technologies). Quantitative PCR (qPCR) was conducted using Taqman system QuantiTect Probe PCR Kit (Qiagen, Hilden, Germany) on ViiA7 Real-Time PCR system (Life Technologies) according to the manufacturer's instructions. Similar results were obtained in two independent experiments. The qPCR primers for survivin were: forward, 5′-AAGAACTGGCCCTTCTTGGA, reverse, 5′-CAACCGGACGAATGCTTTT, probe, 5′-6-FAM-CCAGATGACGACCCCATAGAGGAACA-TAMRA. Actin was used as an internal standard control.

### Cells extracts, western blots and antibodies

To prepare whole-cell extracts for immunoblotting analysis, cells were lysed in RIPA buffer (1% NP-40, 150 mM NaCl, 5 mM EDTA, 0.5% sodium deoxycholate, 50 mM Tris-HCl pH 7.5, 0.1% SDS) with added protease and phosphatase inhibitors 1 mg/ml leupeptin, aprotinin and pepstatin, COMPLETE (Roche Diagnostic, Mannheim, Germany) was added as recommended by the supplier. The buffer was supplemented with PhosStop (Roche) and 1 mM phenylmethylsulfonylfluoride. Equal amounts of protein were loaded on 10–12% SDS-polyacrylamide gels and transferred onto PVDF membranes after electrophoresis. Blots were incubated with primary and horseradish peroxidase-conjugated secondary antibodies. Chemiluminescent detection was used. The antibodies for western blots: anti-survivin (D-8) was purchased from Santa Cruz Biotechnology (Dallas, TX, USA), anti-SRC and anti-BCL2 were from Cell Signaling Technology (Danvers, MA, USA) and *β*-actin (AC-74) from Sigma-Aldrich (St. Louis, MO, USA). Anti-GLI2 (C-10) antibody was from Santa Cruz Biotechnology or Biorbyt (San Francisco, CA, USA). Anti-FLAG antibody (M2) was from Sigma.

### Transfections, luciferase promoter-reporter assay, colony formation

Transient cell transfections of the promoter–reporters (Figures 2 and 3) were performed on 12-well plates by using transfection reagents LipoJet or PolyJet (SignaGen Laboratories, Rockville, MD, USA). The pRSV-Renilla luciferase expression vector was cotransfected to monitor transfection efficiency. pGL3basic vector (Promega, Madison, WI, USA) was used as a control promoter. Expression vectors were cotransfected as indicated in the figures. Cell lysate was used for dual luciferase assays (Promega) performed as recommended by the supplier's instructions on a Turner Designs 20/20 luminometer (Promega). Data were normalized to *Renilla* luciferase activity (internal control) as arbitrary units. The inactive compound structurally similar to cyclopamine (tomatidine) gave the same results as vehicle (not shown). Thus, only vehicle was used as a control in all experiments with cyclopamine. Statistical analysis of luciferase values was performed using a two-tailed unpaired Student's *t-*test. Colony outgrowth assays were carried out by seeding the cells in 12-well plates. After GANT61 treatment for the indicated time period the cells were stained by crystal violet.

### Plasmids and site-directed mutagenesis

pGL3-PTCH1 was obtained from Prof. Aberger, Salzburg, Austria. 12xGLI-TK-Luc plasmid was a gift from Prof. R Toftgard, Karolinska Institutet, Sweden. All versions of the survivin promoter and its mutants have been cloned as *Xho*I–*Hin*dIII inserts in the pGL3basic plasmid. The following survivin promoter–reporter plasmids were generated (numbering is related to the start of translation, +1): −1814+57, −990+57, −1814−10, −990−10, −1814−10Δ(−319−60) and −990−10Δ(−319−60). Further, single-site mutants were prepared by two-step PCR mutagenesis using Phusion DNA polymerase (Fisher Scientific, Pittsburgh, PA, USA). Original GLI1 (GLI K12, #16419), GLI2 (pCS2-MT GLI2 FL, #17648), ΔNGLI2 (pCS2-MT GLI2 delta N, #17649) and GLI3 (GLI3 bs-2, #16420) were purchased from non-profit plasmid repository Addgene (Cambridge, MA, USA). Their coding sequences were amplified by PCR and cloned into the pcDNA3.1 expression vector or to the pFLAG-CMV-4 background to obtain FLAG-tagged GLI proteins for the use in experiments shown in Figure 3c. PCR was used for cloning all GLIs to the final plasmids. pCMV-Sp1 was obtained from Addgene (#12097), pCMV6-XL5-NfkappaB from Origene (Rockville, MD, USA). All final plasmids were verified by sequencing (GATC Biotech, Constance, Germany).

### Chromatin immunoprecipitation

A549 cells were transfected with the pcDNA3-ΔNGLI2 expression plasmid. After 2 days cells were fixed with 1% formaldehyde, incubated with glycine solution and washed four times with PBS. The cell extracts were isolated and processed according to instructions of the ChIP-IT High Sensitivity Kit (Active Motif, Carlsbad, CA, USA). As a positive control, anti-acetylated histone H4 antibody was used (Millipore, Billerica, MA, USA). Negative controls were buffer, rabbit or mouse non-immune IgG. To detect GLI2 bound on the promoter, mouse anti-GLI2 (C-10) (Santa Cruz Biotechnology; sc-271786) and rabbit anti-GLI2 (Abcam, Cambridge, UK; ab26056) were used. For the detection of ChIP-generated DNA, real-time PCR was performed by the QuantiTect SYBR Green PCR Kit (Qiagen). The amplification has been performed with two alternative primer pairs. The primers used for the amplification were sense 5′-TTTGTCCTTCATGCCCGTCT, antisense 5′-TGTAGAGATGCGGTGGTCCT, or the same sense primer and a different antisense, 5′-GCGGGGCATGTCGGGA. Both amplifications gave similar results.

### Tumor xenografts

To investigate whether the GANT61 regulates HH/GLI-induced growth *in vivo*, melanoma cell lines SK-MEL-3 and 501mel were engrafted subcutaneously into athymic nude mice (strain CD-1, 1 million of cells per single site). Cells were resuspended in Matrigel (Becton Dickinson, Franklin Lakes, NJ, USA) and inoculated in one site of the right lateral flank of 4- to 5-week-old female mice (Jackson Laboratories, Sacramento, CA, USA). Subcutaneous tumor size was measured three times a week with a caliper and tumor volumes were calculated. GANT61 was administered three times a week intratumorally for 2 weeks. After ending the experiment, the fresh tumor tissue was used for western blot and analyzed by immunochemistry. The tumor results were analyzed at the Institute of Biophysics and Informatics, Charles University in Prague, 1st Faculty of Medicine. The experiment was approved by the Commission for Experimental Animals of the medical faculty and was in accordance with the national guidelines and regulations. Statistical analyses were performed using a two-tailed unpaired Student's *t-*test.

### Immunohistochemistry and immunofluorescence

Paraffin-embedded sections were obtained from the Institute of Pathology, Charles University in Prague, 1st Faculty of Medicine. Experiments were carried out with the approval of the Ethics Committee of the General University Hospital. Parallel tissue sections were stained with survivin and GLI2 primary antibodies purchased from GeneTex (Irvine, CA, USA). The detection of antigen–antibody complexes was performed using EnVision+ avidin-biotin detection system (Dako, Glostrup, Denmark). Sections were independently examined by two pathologists. Tissues were scored on a scale of 0 (negative) to 4 (highly positive) based on the intensity of staining and evaluated for statistical significance by Student's *t*-test. Only highly positive areas (scored 4), the examples of which are shown in Figure 6b, showed positive correlation in the whole sections. Thirty-five tumors of various types were examined, including non-small-cell lung carcinomas, primary melanomas, ovarian carcinomas and tubal intraepithelial carcinomas. Mice tumors were stained only for survivin. Colocalization by double immunofluorescence has been carried out on the frozen tissue sections after acetone fixation (10 min, −80 °C). Simultaneously added primary antibodies against GLI2 and survivin were as for IHC and labeled secondary antibodies were purchased from Abcam.

### Statistical analysis

Statistical significance (*P*-values) was calculated by two-tailed Student's *t*-test. S.E. values are indicated in graphs as bars in each column in the luciferase and real-time PCR assays. In the figures, values of *P*<0.05 are marked by an asterisk, data that are not significant (*P*>0.05) are labeled #. Values with *P*<0.01 are not marked. Western blots were quantified by ImageJ software. In IHC and immunofuorescence sections, chosen highly positive structures were quantified and correlated by FIJI software.

## Abbreviations

DEC1: differentiated embryo-chondrocyte 1
HH/GLI: Hedgehog–GLI signaling pathway
GLI: glioma-associated oncogene homolog
KLF5: Kruppel-like factor 5
Shh: sonic hedgehog
STAT3: signal transducer and activator of transcription 3
