# Supplementary material for: Survivin, a novel target of the Hedgehog/GLI signaling pathway in human tumor cells
Source: Cell Death Dis. 2016 Jan 14;7(1):e2048–. doi: 10.1038/cddis.2015.389 (PMC4816174; doi:10.1038/cddis.2015.389)
Supplement: Supplementary Information [file cddis2015389x1.doc]

# Figure and Table Legends to Supplementary Material

**Table S1** Human cell lines used in this study were purchased from American Type Culture Collection except for marked cell lines (*): 501mel cell line was obtained from Yale University (Dr. R. Halaban), cell lines DOR, BEU and HBL are from Free University of Brussels (Dr. G. Ghanem). PA-TU-8902 cell line was obtained from Deutsche Sammlung von mikroorganismen und Zellkulturen (DSMZ), Germany. All cell lines are certified (ATCC) or authenticated in the original organization. More than half of the cell lines are primary or metastatic melanomas and small cell lung cancers. Primary melanomas are WM35 and WM1552C.

**Figure S1** Increasing dose dependent effect of cyclopamine and GANT61 on the survivin promoter in SK-MEL-3 cell line shows increased survivin promoter suppression at higher doses of cyclopamine and GANT61. The cells were transfected with the survivin promoter, washed, and the agents were added for the last 24hrs of the transfection experiment.

**Figure S2 (a)** Cells (A549) were transfected increasing amounts of the indicated plasmid construct. Dual luciferase assay was performed after 48h. No or minimal increase was observed with GLI1 and GLI3 plasmids. SE values are indicated on each column. *, statistically significant (P<0.05), #, statistically not significant. If no mark is shown, the values are statistically significant at P<0,01 (all statistics is related to the control)

**(b)** Bordered Figure shows the comparison of the activity of GLI1 and GLI2 with NFκB and Sp1, which also activate the survivin promoter.

**Figure S3** Cell lines showing minimal or no effect in survivin levels after GANT61 treatment. Cell lines in which the survivin expression was not influenced (or the changes were only infinitesimal) are depicted. Survivin was not expressed in Malme-3M and NCI-H596 cell lines. The conditions of GANT61 treatment and W. blot detection were essentially the same as in Figure 4a.

**Figure S4** Linear regression depicting a weak correlation (P<0.05) between survivin protein abundance and survivin mRNA after GANT treatment. Quantitation of protein data were obtained from 20 μM GANT61 shown in Figure 4a with the ImageJ software. RNA levels data were from the real time PCR experiment (Figure 4b). Only cell lines used for real time PCR are used in the statistics. The parametric Pearson correlation coefficient is shown.

**Figure S5** Linear regression documenting that the survivin protein abundance and GLI protein levels do not correlate P>0.05). Quantitation of protein for survivin data was obtained from controls (0 μM GANT61) shown in Figure 4a and GLI2 from Western blots (not shown) by the ImageJ software. All 40 cell lines used were analysed. The parametric Pearson correlation coefficient is shown.

# Figure S6 Survivin protein degradation. H1299 cells have increased survivin protein level after the addition of proteasome inhibitor MG132 during the GLI activity is blocked by GANT61, indicating GLI-independent protection of survivin degradation. Survivin decrease in A427 cells is justified by an RNA decrease (Figure 4b). 501mel cells react poorly to GANT61. In SK-MEL-3 cells, MG132 has not increased the survivin level at the end of GANT61 incubation, suggesting the degradation of survivin protein is protected by the activity of GLI factors. MG, MG132 (20μM); GANT, GANT61 (20μM). Cells were treated with GANT61 for 24h and MG132 was added for the last 6h of incubation. Cells were then lysed in RIPA buffer and analysed.

**Figure S7** Tumor xenografts of melanoma cells. (**a,b**) Tumor apperance in controls and groups treated with GANT61 in two indicated cell lines. One of two independent experiments is shown (four mice per condition in each experiment; only 3 mice are shown because in each group one tumor did not outgrow or regressed, respectively; not shown). Similar results were obtained in both experiments. Statistical analysis was performed by a two-tailed unpaired Student’s test. For 501mel cells P<0.05 values were obtained. (**c,d**) Western blots from the tumor tissue showing survivin expression. (**e**) Cell proliferation in vitro. Cell were untreated (C) or treated with 20μM GANT61 (G) for 4 days, fixed and stained. Quantification data obtained by ImageJ software are shown in each window. The growth decrease in each cell line was statistically significant (P<0.01). (**f**) Survivin expression in immunohistochemical sections of tumors from control group of mice with 501mel tumors (scored 4), SK-MEL-3 tumors (scored 3) and mice treated with GANT61 (scored 0-1). Scarcely scattered survivin positive cells were seen in tumors from GANT61 treated animals. Bar, 100 μm. **(g)** Growth curves of tumors from SK-MEL-3 cells. The results were not significant (P>0.05) due to one tumor from the GANT61-treated group reached the final volume similar to the two control tumors. **(h)** Growth curves of tumors from inoculated 501mel cells. The tumors did not diminish, which correlated with in vitro resistance of these cells to GANT61 (correlation: P<0.05). Please note different Y-axis values. ▲, tumor 1; ⏺, tumor 2; o , tumor 3.
