# Supplementary material for: Survivin, a novel target of the Hedgehog/GLI signaling pathway in human tumor cells
Source: Cell Death Dis. 2016 Jan 14;7(1):e2048–. doi: 10.1038/cddis.2015.389 (PMC4816174; doi:10.1038/cddis.2015.389)
Supplement: Supplementary Table S1 [file cddis2015389x2.doc]

| Cell line | Tumor type | Cell line | Tumor type |
| --- | --- | --- | --- |
| Malme-3M | Malignant melanoma | NCI-H378 | Small cell lung cancer |
| MeWo | Malignant melanoma | NCI-H1299 | Non-small cell lung cancer |
| SK-MEL-1 | Malignant melanoma | A549 | Non-small cell lung carcinoma |
| SK-MEL-2 | Malignant melanoma | A-427 | Non-small cell lung carcinoma |
| SK-MEL-3 | Malignant melanoma | SK-MES-1 | Squamous cell lung carcinoma |
| SK-MEL-5 | Malignant melanoma | NCI-H596 | Adenosquamous lung carcinoma |
| SK-MEL-28 | Malignant melanoma | HCT 116 | Colorectal cell carcinoma |
| RPMI-7951 | Malignant melanoma | LoVo | Colorectal adenocarcinoma |
| 501mel* | Malignant melanoma | SW480 | Colorectal adenocarcinoma |
| DOR* | Malignant melanoma | C-33 A | Cervical carcinoma |
| BEU* | Malignant melanoma | HeLa S3 | Cervical adenocarcinoma |
| HBL* | Malignant melanoma | SK-N-SH | Neuroblastoma |
| WM35 | Malignant melanoma | SK-N-MC | Neuroepithelioma |
| WM1552C | Malignant melanoma | T98G | Glioblastoma multiforme |
| NCI-H69 | Small cell lung cancer | MIA PaCa-2 | Pancreatic carcinoma |
| NCI-H82 | Small cell lung cancer | PA-TU-8902* | Pancreatic adenocarcinoma |
| NCI-H146 | Small cell lung cancer | BxPC3 | Pancreatic adenocarcinoma |
| NCI-H196 | Small cell lung cancer | Jurkat, cl. E6-1 | Acute T cell leukemia |
| NCI-H209 | Small cell lung cancer | SW-13 | Adrenal gland primary small cell ca. |
| NCI-H345 | Small cell lung cancer | Hep-G2 | Hepatocellular carcinoma |

**SUPPLEMENTARY TABLE S1, Vlčková et al.**
