# Supplementary figures and images for: Survivin, a novel target of the Hedgehog/GLI signaling pathway in human tumor cells
Source: Cell Death Dis. 2016 Jan 14;7(1):e2048–. doi: 10.1038/cddis.2015.389 (PMC4816174; doi:10.1038/cddis.2015.389)

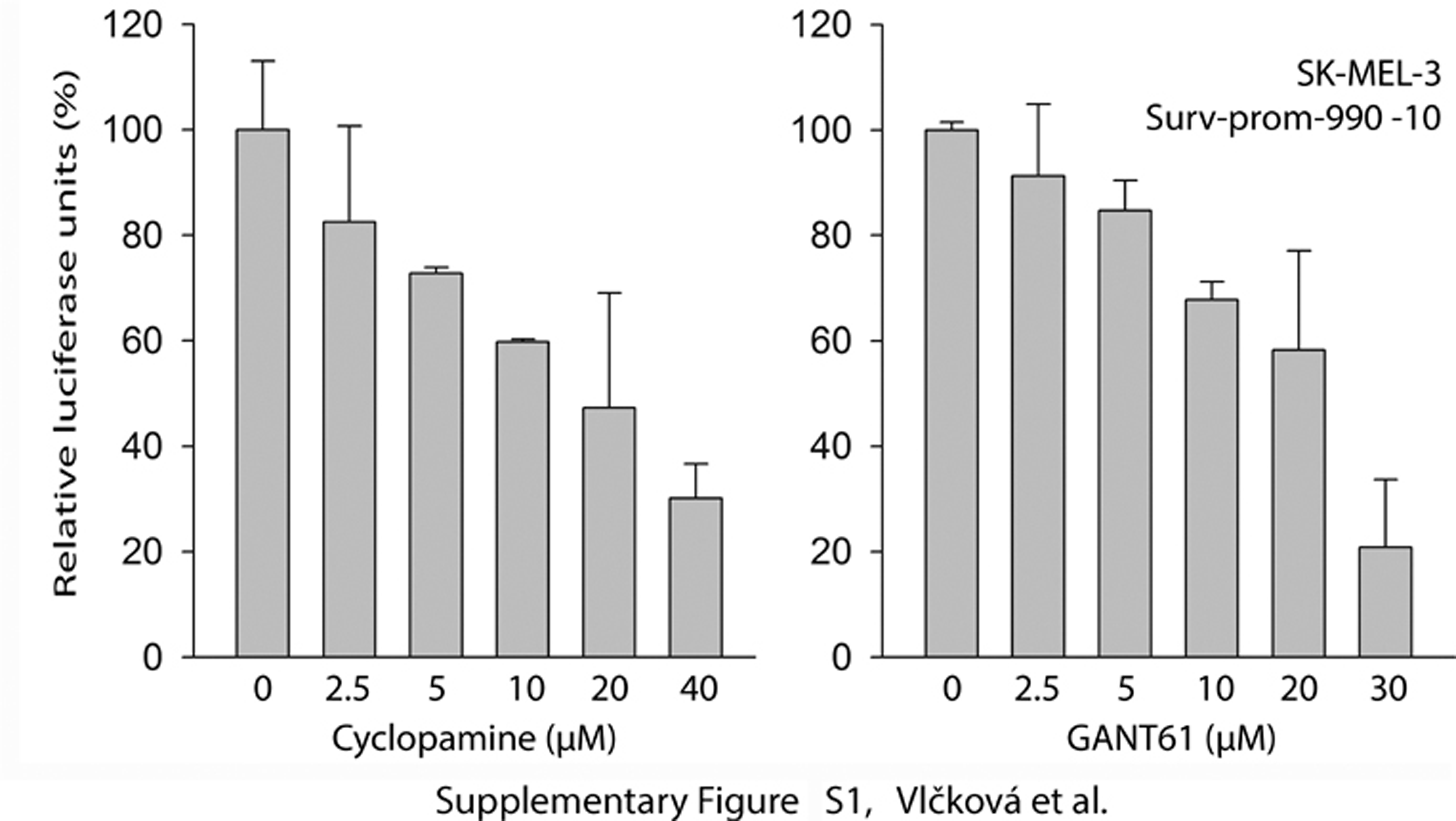

Supplement: Supplementary Figure S1 [file cddis2015389x3.tif]

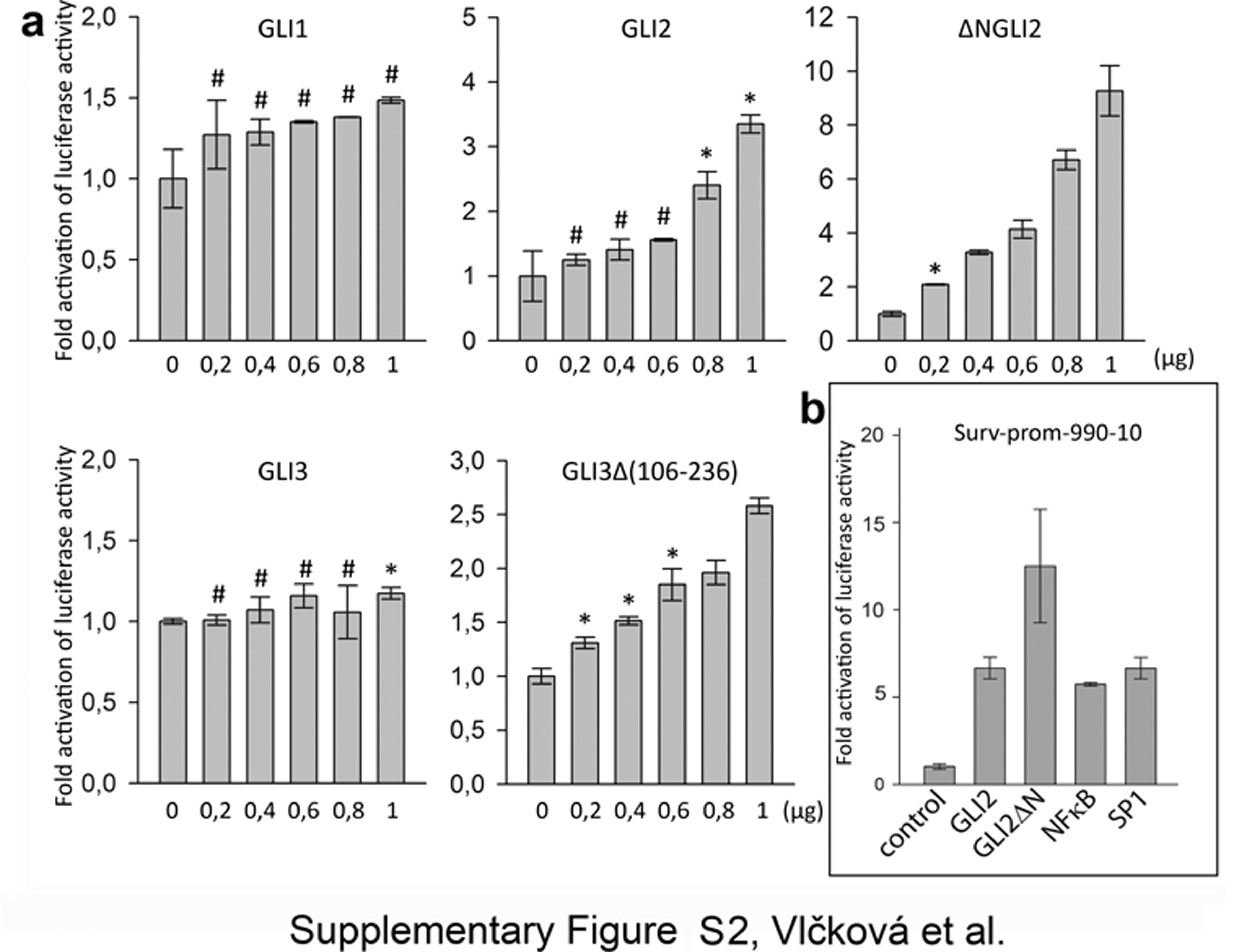

Supplement: Supplementary Figure S2 [file cddis2015389x4.tif]

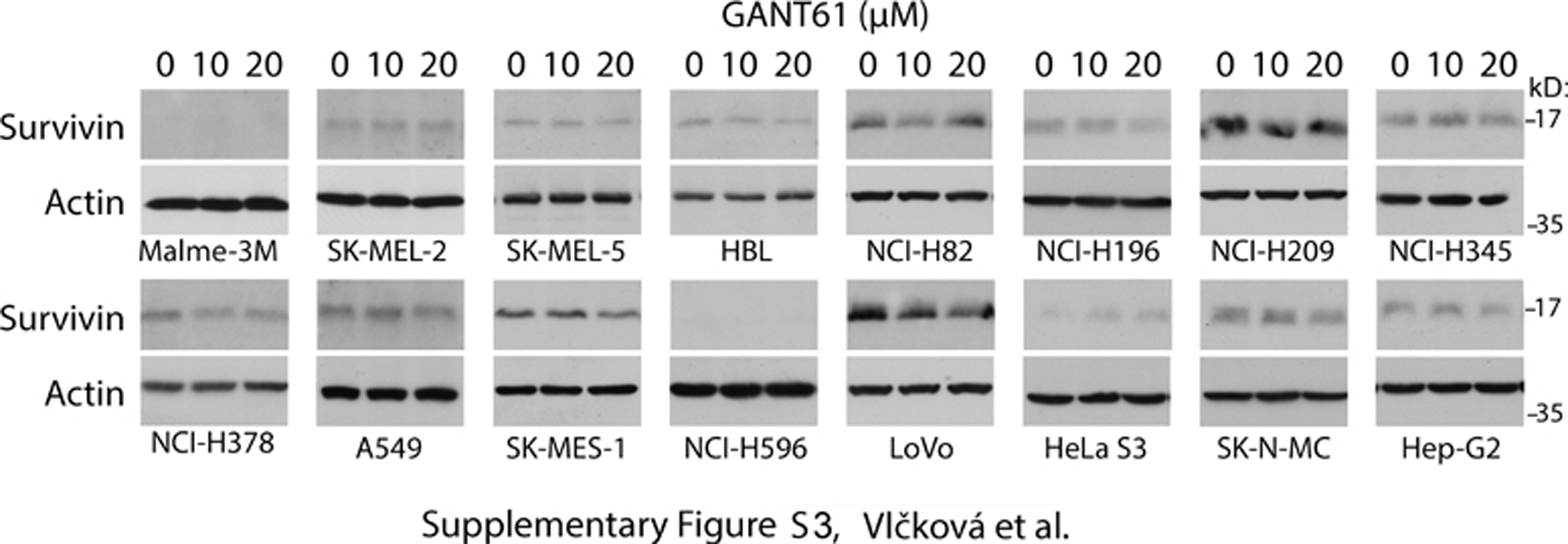

Supplement: Supplementary Figure S3 [file cddis2015389x5.tif]

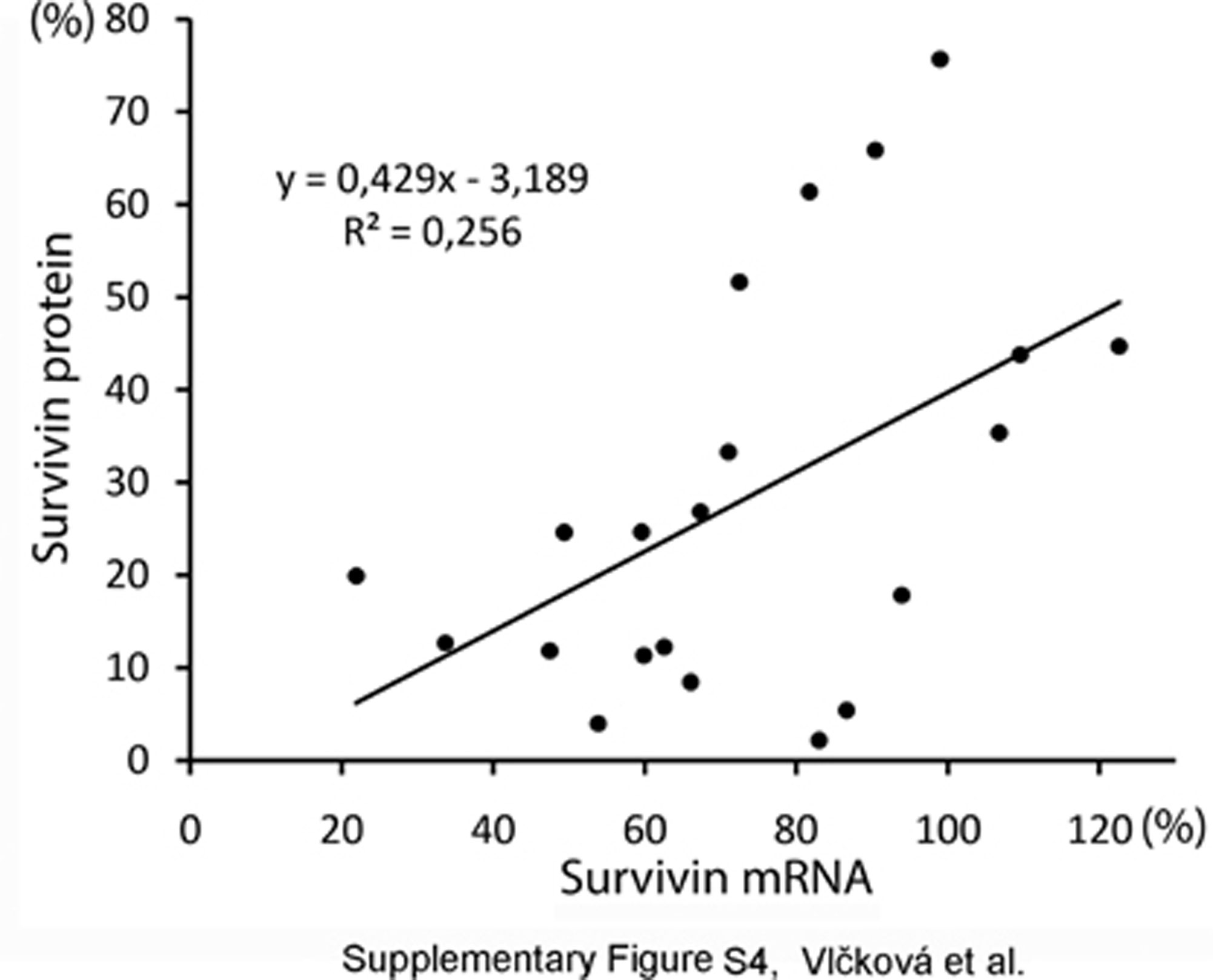

Supplement: Supplementary Figure S4 [file cddis2015389x6.tif]

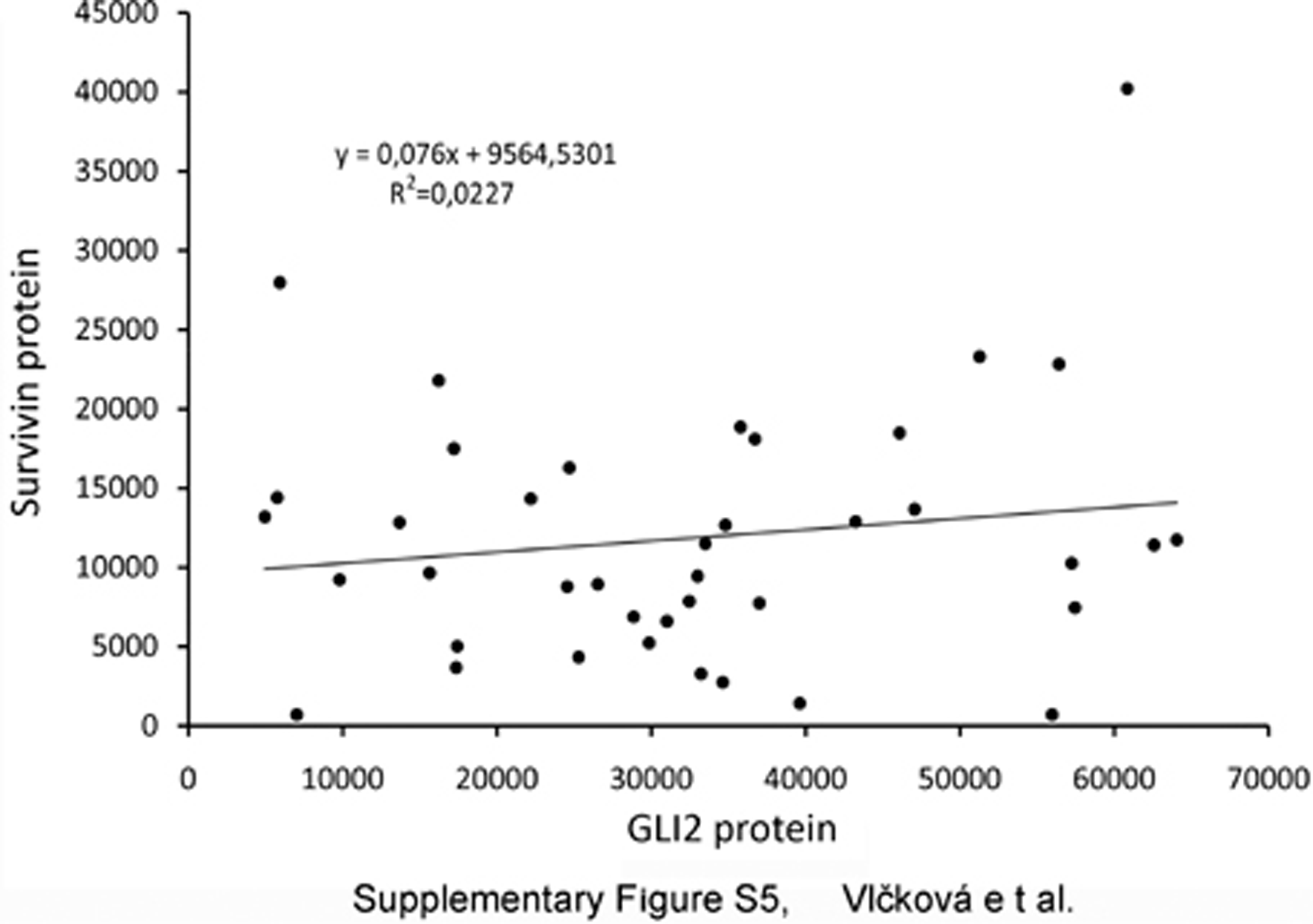

Supplement: Supplementary Figure S5 [file cddis2015389x7.tif]

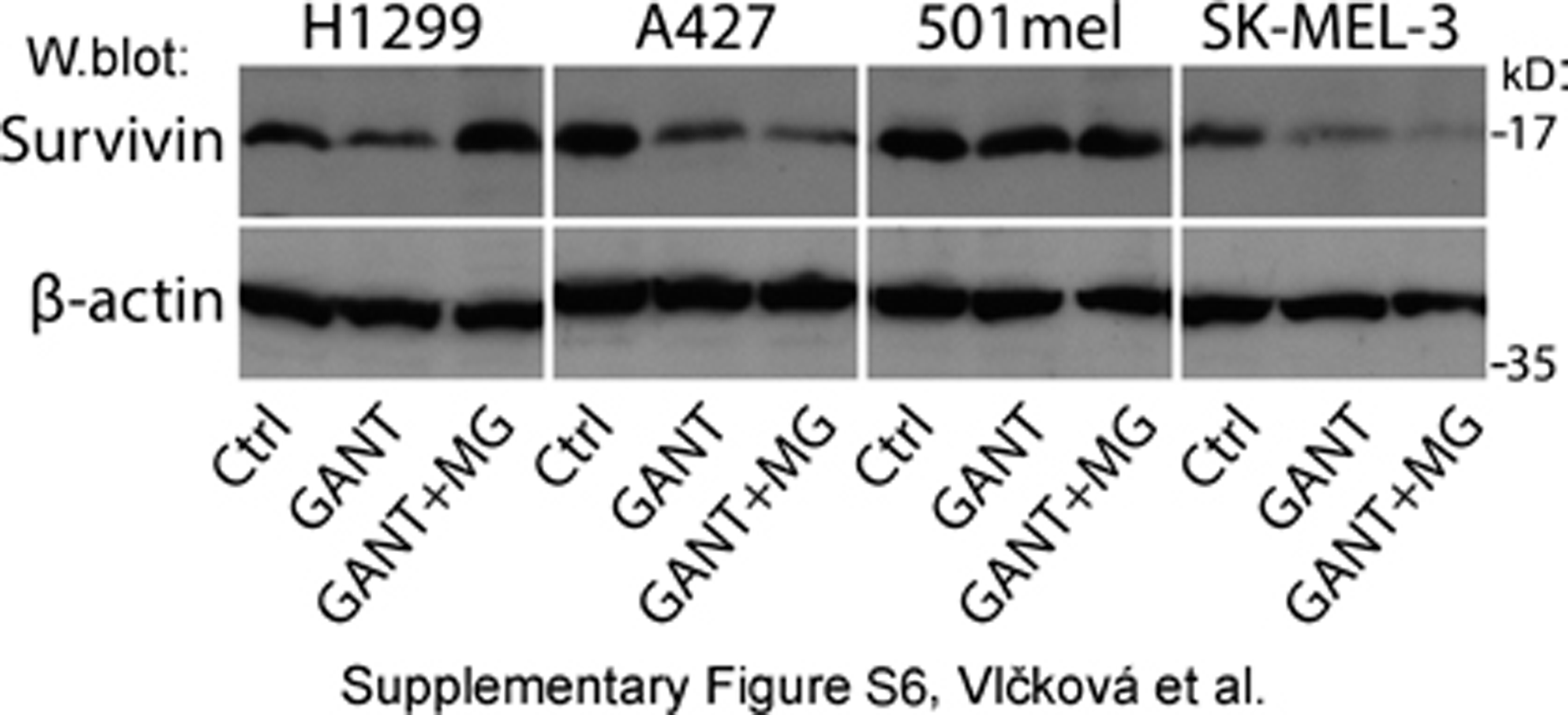

Supplement: Supplementary Figure S6 [file cddis2015389x8.tif]

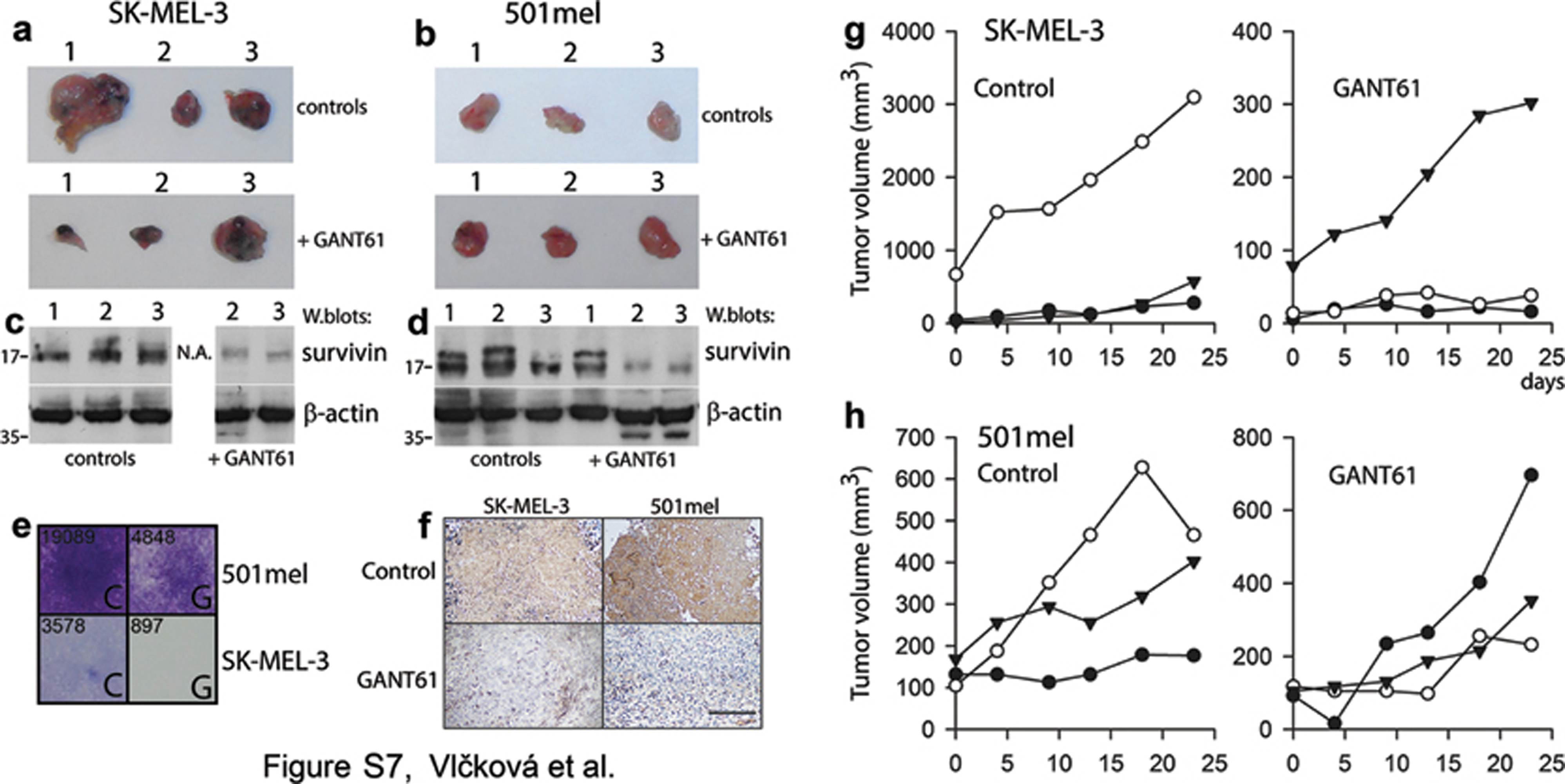

Supplement: Supplementary Figure S7 [file cddis2015389x9.tif]
